# Verification of Candidate SNP Effects Reveals Two QTLs on BTA7 for Beef Marbling in Two Japanese Black Cattle Populations

**DOI:** 10.3390/genes13071190

**Published:** 2022-07-01

**Authors:** Shinji Sasazaki, Raito Yamamoto, Shintaro Toyomoto, Hina Kondo, Takayuki Akiyama, Namiko Kohama, Emi Yoshida, Fuki Kawaguchi, Kenji Oyama, Hideyuki Mannen

**Affiliations:** 1 Laboratory of Animal Breeding and Genetics, Graduate School of Agricultural Science, Kobe University, Kobe 657-8501, Japan; raiphil0802@icloud.com (R.Y.); toyo.toyo.soccer.10@gmail.com (S.T.); proton1727@yahoo.co.jp (H.K.); kawaguchi@koala.kobe-u.ac.jp (F.K.); mannen@kobe-u.ac.jp (H.M.); 2 Hokubu Agricultural Technology Institute, Hyogo Prefectural Technology Center for Agriculture, Forestry & Fisheries, Asago 669-5254, Japan; Takayuki_Akiyama@pref.hyogo.lg.jp (T.A.); Namiko_Kohama@pref.hyogo.lg.jp (N.K.); 3 Hyogo Prefectural Technology Center for Agriculture, Forestry and Fisheries, Kasai 679-0198, Japan; Emi_Yoshida@pref.hyogo.lg.jp; 4 Food Resources Education & Research Center, Kobe University, Kasai 675-2103, Japan; oyama@kobe-u.ac.jp

**Keywords:** GWAS, beef marbling, Japanese Black cattle, meat quality, *ICAM1*

## Abstract

In our previous study, we used genome resequencing to detect all candidate polymorphisms within a quantitative trait loci (QTL) region for beef marbling reported previously at 10–30 Mbp on bovine chromosome 7, and we selected 6044 polymorphisms as candidate quantitative trait nucleotides (QTNs). In the present study, we aimed to identify quantitative trait genes (QTGs) and QTNs in this QTL region by verifying the effect of SNPs on beef marbling in two Japanese Black cattle populations using a Dynamic Array integrated fluidic circuit. In total, 96 selected SNPs were genotyped in 441 and 529 animals in Hyogo and Miyazaki cattle populations, respectively. The most significant *p*-values were detected in a SNP in a splice region of *ALDH7A1* (SNP93_*ALDH7A1*; *p* = 3.46 × 10^−5^) in Hyogo cattle and a missense polymorphism of intercellular adhesion molecule-1 (*ICAM1*) (SNP37_*ICAM1*; *p* = 3.33 × 10^−4^) in Miyazaki cattle. Interestingly, SNP93_*ALDH7A1* was not significant (*p* = 0.459) in Miyazaki cattle, and SNP37_*ICAM1* showed a weakly significant association (*p* = 0.043) in Hyogo cattle. Thus, each population would likely have different QTGs and QTNs for beef marbling in the QTL region. In the Hyogo population, it was not possible to determine the accurate range of the linkage disequilibrium (LD) block in LD block analysis because of a strong LD structure throughout the assessed region. In Miyazaki cattle, however, an LD block containing SNP37_*ICAM1* had a range of 15.8–16.1 Mbp, suggesting that QTNs would be located within this region. The functions of 19 genes in the LD block were investigated. *ICAM1* is known to play an important role in adipocyte differentiation; given this function and the effect of amino acid substitution, SNP37_*ICAM1* was identified as a promising candidate QTN for beef marbling. Further research on the effect of SNP37_*ICAM1* on adipocyte differentiation is expected to provide insights into the mechanism underlying beef marbling formation.

## 1. Introduction

Japanese Black is the main breed of beef cattle in Japan, and beef marbling is abundant in the meat of this breed [1]. Beef marbling has been improved in Japanese Black cattle over time, but the heritability of the beef marbling standard (BMS), an indicator of beef marbling in Japan, has remained high at 0.5–0.6 [2], suggesting that beef marbling-related genetic variation still exists. Some researchers have searched for quantitative trait nucleotides (QTNs) and quantitative trait genes (QTGs) for beef marbling, and some beef marbling-associated polymorphisms have been reported [3,4]. However, the primary gene(s) responsible for controlling beef marbling remains unknown.

In a previous study, we investigated the quantitative trait loci (QTLs) for beef marbling by performing a genome-wide association study (GWAS) in Japanese Black cattle in Hyogo prefecture, and we identified a candidate region (10–30 Mbp) in bovine chromosome 7 (BTA7) [5] that was consistent with previous QTL reports of beef marbling-related traits in various breeds and populations [6,7,8]. These results suggested that major QTGs and QTNs for beef marbling would be located within this region. Therefore, in a subsequent study, we performed genome resequencing to detect all polymorphisms in this candidate region [9]. From the 127,090 polymorphisms detected, we identified 6044 SNPs based on gene annotation and linkage disequilibrium (LD) with the most significant SNP (i.e., the “top” SNP) in the GWAS. One of the SNPs, a nonsynonymous substitution (K81M) of *SLC27A6*, was identified as a putative candidate polymorphism, and the effect of this SNP on beef marbling was verified. Indeed, *SLC27A6* K81M had a more significant *p*-value (*p* = 0.0009) than the top SNP (*p* = 0.0049), suggesting that it was a promising candidate QTN for beef marbling. However, additional verification of the remaining candidate polymorphisms is required to identify all candidate QTNs and QTGs. Furthermore, verification of the effects of these SNPs using an additional population will also help validate the QTNs.

Therefore, in the current study, we used a Dynamic Array integrated fluidic circuit (IFC) to verify the effects of the remaining candidate polymorphisms in two cattle populations from Hyogo and Miyazaki prefectures, with the aim of identifying QTGs and QTNs in the aforementioned QTL region.

## 2. Materials and Methods

All experiments in the current study were conducted according to the Kobe University Animal Experimentation Regulations. We used two Japanese Black cattle populations bred in Hyogo and Miyazaki prefectures. The Hyogo population comprised 441 cattle (352 steers and 89 heifers), which were produced from 7 sires and slaughtered at 31.69 ± 1.24 months of age on average. The Miyazaki population comprised 529 cattle (477 steers and 52 heifers), which were produced from 6 sires and slaughtered at 29.10 ± 1.62 months of age on average. The average BMS scores in the Hyogo and Miyazaki populations were 5.66 ± 1.76 and 6.08 ± 1.96, respectively. Genomic DNA was extracted from 50-mg longissimus cervicis muscle samples using a standard phenol–chloroform method. An ethical review and approval for use of these samples were not required because the samples were collected from cattle in slaughterhouses that were slaughtered for sale on the market.

In total, 96 SNPs were selected from 6044 candidate SNPs based on gene functions and annotations for each polymorphism (Supplementary Table S1). Information on genomic position is based on the DNA reference sequence of the UMD3.1.1 assembly (GenBank accession no.: GCA_000003055). The primer sets used to genotype the 96 SNPs were designed by Fluidigm Corporation (South San Francisco, CA, USA).

Genotyping was performed using a Fluidigm Biomark HD system (Fluidigm Corporation, South San Francisco, CA, USA). First, preamplification was performed using specific target amplification and locus-specific primers according to the manufacturer’s instructions. Second, sample mixes for 96 individuals and assay mixes for 96 SNPs were prepared for genotyping using the 96.96 IFC. The sample and assay mixes contained the preamplification products for each individual and allele-specific primer 1 and 2 for each SNP, respectively. The composition of the sample and assay mixes followed that described in the manufacturer’s protocol. Each 5-μL sample mix and 4-μL assay mix was separately inletted into the IFC. Amplification was performed using the following thermocycling protocol: 70 °C for 30 min; 25 °C for 10 min; 95 °C for 5 min; four cycles of 95 °C for 15 s, 64 °C to 61 °C (decreasing by 1 °C per cycle) for 45 s, and 72 °C for 15 s; and 34 cycles of 95 °C for 15 s, 60 °C for 45 s, and 72 °C for 15 s. Fluorescence data collected from the Fluidigm Biomark HD system were analyzed using Fluidigm SNP Genotyping Analysis software to classify the samples into three genotypes based on the fluorescence intensities of FAM and HEX.

ANOVA was used to assess the effects of all genotyped SNPs on BMS. The analytical model for the Hyogo population included the effect of sire, sex, slaughter year, slaughter month, genotype, and linear and quadratic covariates for the age at slaughter. A similar analytical model was applied to the Miyazaki population. However, the effects of slaughter year and month were removed because they were not statistically significant in the Miyazaki population. We also performed ANOVA in considering the genotypes of SNP93_*ALDH7A1* and SNP37_*ICAM1* as fixed effects in the Hyogo and Miyazaki populations, respectively. Differences between least-squares means for genotypes within a gene were assessed using Tukey–Kramer’s honestly significant difference test. LD blocks were constructed using HAPLOVIEW 4.0 [10].

## 3. Results

### 3.1. Genotyping Using the Fluidigm Biomark HD System

Using the Fluidigm Biomark HD system, the 96 SNPs selected as candidate polymorphisms were genotyped in 441 and 529 animals in the Hyogo and Miyazaki populations, respectively. Information on the 96 SNPs and genotyping results for each population are shown in Table S1. SNPs for which genotyping was difficult because of weak signals were excluded from further analysis. Thus, of the 96 selected SNPs, 78 and 77 SNPs in the Hyogo and Miyazaki populations, respectively, were successfully genotyped. Of these, 11 and 12 SNPs in the Hyogo and Miyazaki populations, respectively, were excluded from further analysis because of the lack of polymorphisms.

### 3.2. Effect of Genotyped SNPs on BMS in Two Populations

**Figure 1:**
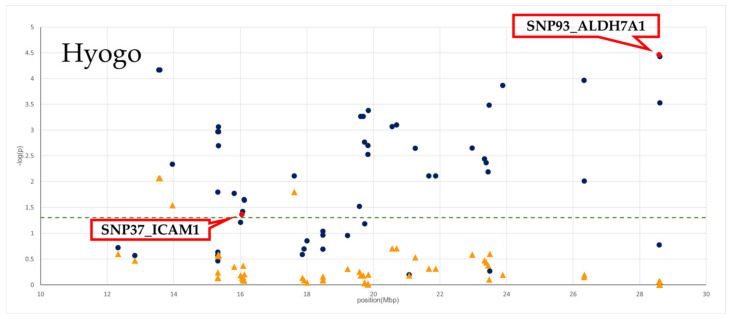

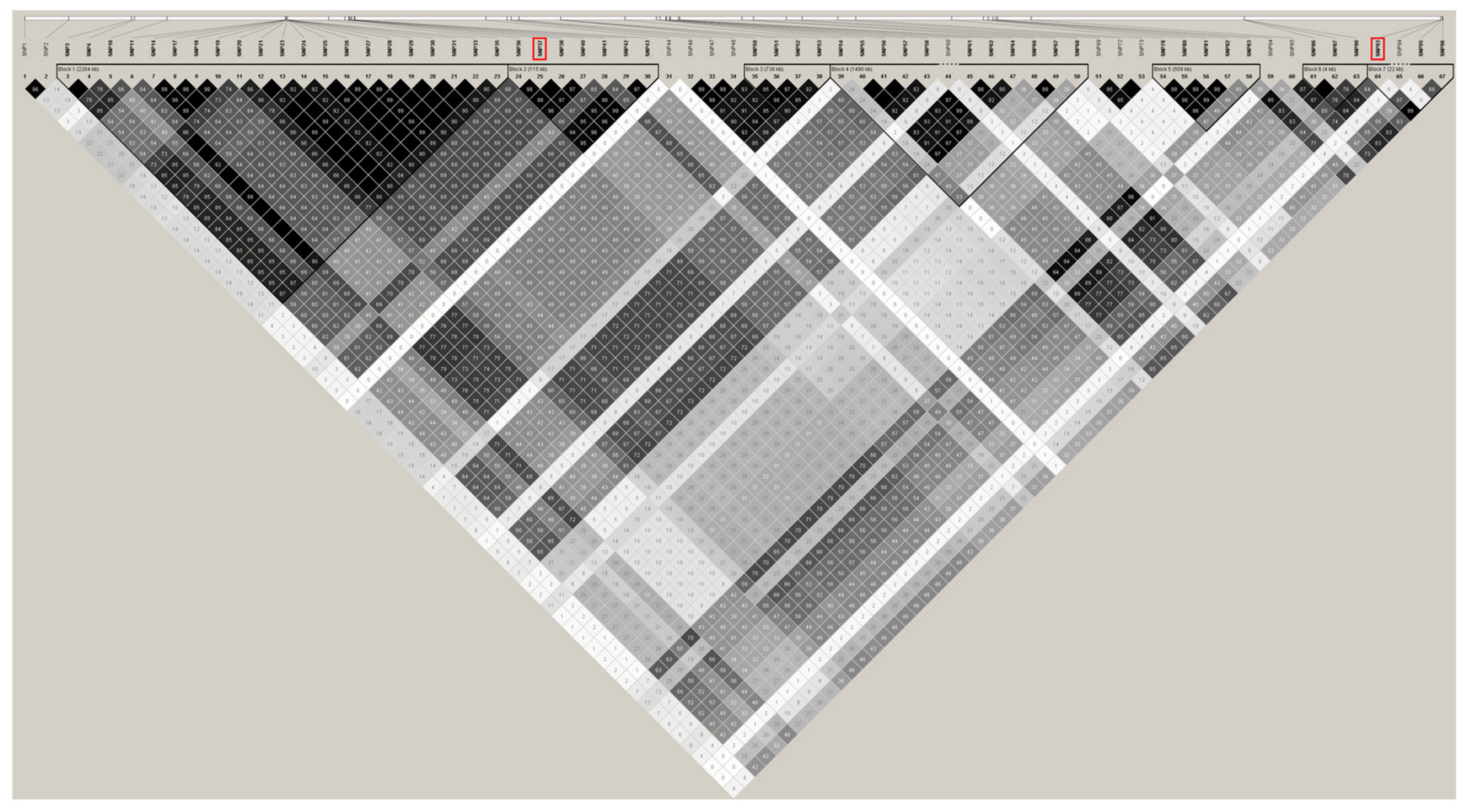
SNPs’ significance plot for BMS and LD block from 10 to 30 Mbp on BTA7 in Hyogo population. The *x*-axis indicates chromosome 7 positions in base pairs, and the *y*-axis indicates log–inverse *p*-values. Significance of SNPs is represented as blue circle for independent SNP associations and as yellow triangle for SNP associations estimated by fitting SNP93_*ALDH7A1* as a fixed effect in the model. The significance of SNP93_*ALDH7A1* and SNP37_*ICAM1* SNP is represented by red circle. The dashed green line indicates the threshold of the 5% significance level. LD coefficients (*r*^2^) between the SNPs in this region. Black squares indicate *r*^2^ values >0.80, and white and gray indicate *r*^2^ values <0.80. SNP93_*ALDH7A1* and SNP37_*ICAM1* SNP are shown by a red frame.

**Figure 2:**
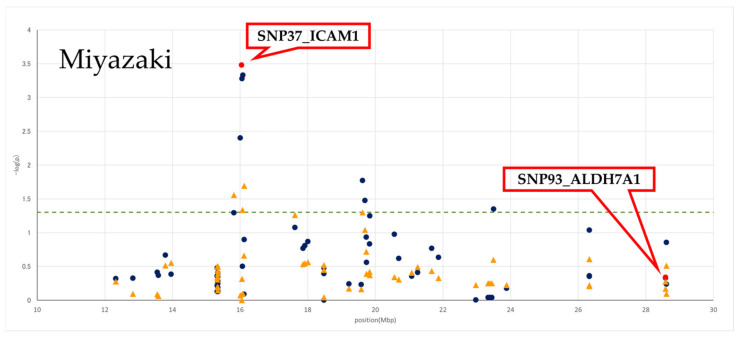

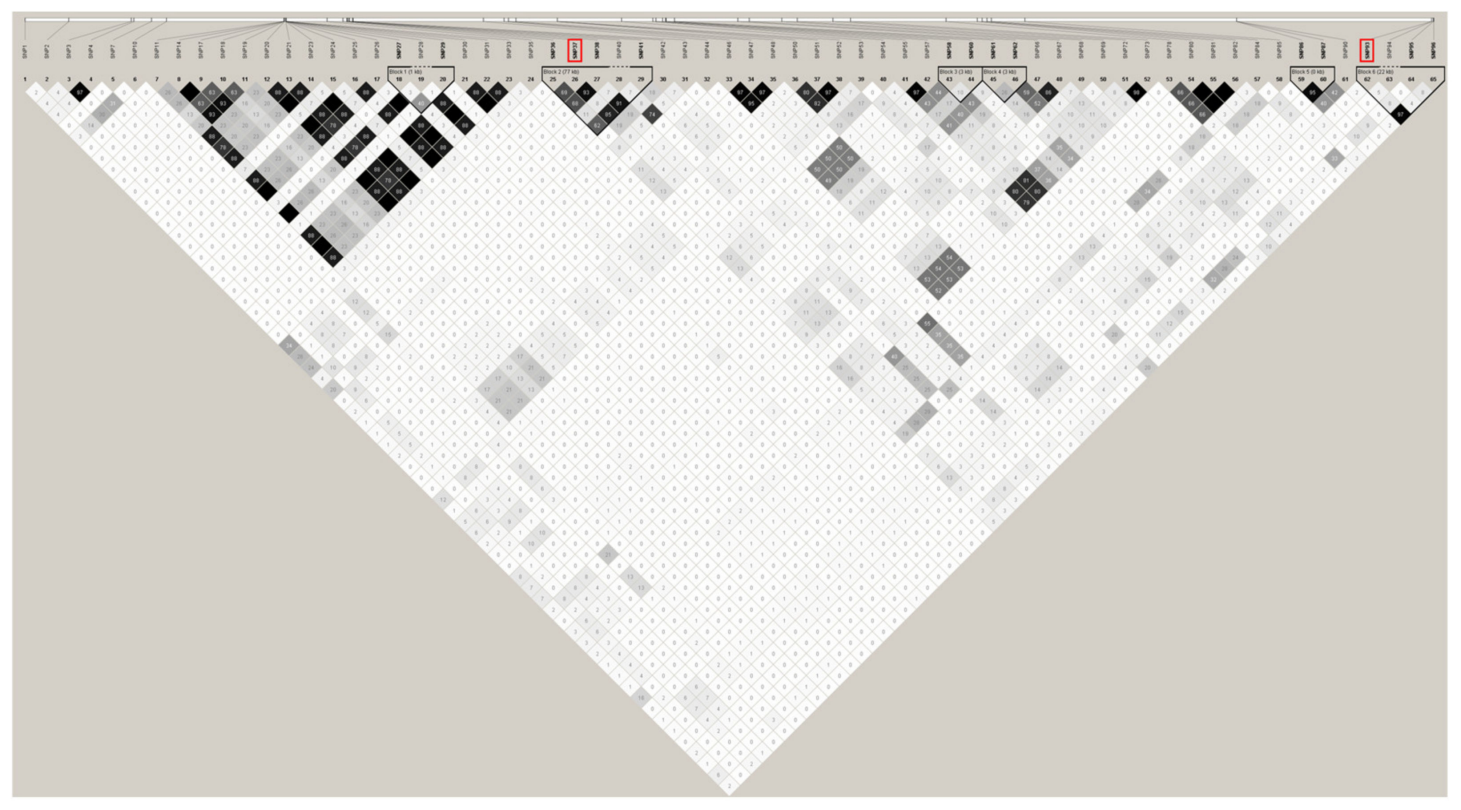
SNPs’ significance plot for BMS and LD block from 10 to 30 Mbp on BTA7 in Miyazaki population. The *x*-axis indicates chromosome 7 positions in base pairs, and the *y*-axis indicates log–inverse *p*-values. Significance of SNPs is represented as blue circle for independent SNP associations and as yellow triangle for SNP associations estimated by fitting SNP37_*ICAM1* SNP as a fixed effect in the model. The significance of SNP93_*ALDH7A1* and SNP37_*ICAM1* SNP is represented by red circle. The dashed green line indicates the threshold of the 5% significance level. LD coefficients (*r*^2^) between the SNPs in this region. Black squares indicate *r*^2^ values >0.80, and white and gray indicate *r*^2^ values <0.80. SNP93_*ALDH7A1* and SNP37_*ICAM1* SNP are shown by a red frame.

The effects of 67 and 65 SNPs on the BMS in the Hyogo and Miyazaki populations, respectively, were assessed using ANOVA (*p*-values and a *p*-value plot are provided in Table S1, Figure 1 and Figure 2). In Hyogo cattle, 48 SNPs from 29 genes were statistically significant (*p* < 0.05), and the most significant *p*-value was detected in an SNP in a splice region of *ALDH7A1* (SNP93_*ALDH7A1*; *p* = 3.46 × 10^−5^). In Miyazaki cattle, seven SNPs from seven genes were statistically significant (*p* < 0.05), and the most significant *p*-value was detected in a missense SNP in the intercellular adhesion molecule-1 (*ICAM1*) (SNP37_*ICAM1*; *p* = 3.33 × 10^−4^).

Considering the genotypes of SNP93_*ALDH7A1* and SNP37_*ICAM1* as fixed effects in each population, *p*-values were also recalculated according to conditioned analysis (also see Table S1, Figure 1 and Figure 2). After conditioning, the associations of the genotyped SNPs in the assessed region were generally decreased in each population, suggesting that the region contained a single QTL.

### 3.3. LD Block Analysis

In each population, LD blocks were constructed using Haploview with the genotypes of 67 and 65 SNPs in the Hyogo and Miyazaki populations, respectively (Figure 1 and Figure 2). In Hyogo cattle, relatively strong LD between the SNPs in the assessed region was observed. Contrastingly, some LD blocks were observed in the Miyazaki population, and SNP37_*ICAM1* was included in a block with a range of 15.8–16.1 Mbp.

### 3.4. Statistical Analysis of the Most Significant SNPs

Table 1 and Table 2 show the gene frequencies and effects on the BMS of SNP93_*ALDH7A1* and SNP37_*ICAM1* in the Hyogo and Miyazaki populations. The minor allele frequencies of SNP93_*ALDH7A1* were 0.24 and 0.13 in Hyogo and Miyazaki cattle, respectively. This SNP was most significant (*p* = 3.75 × 10^−5^) in the Hyogo population but not significant (*p* = 0.4593) in the Miyazaki population. Comparing the least-squares means between genotypes, significant differences were detected among AA, AC, and CC in Hyogo cattle, suggesting that animals with the A allele have a higher BMS score than those with the C allele. In the Miyazaki population, no significant differences were observed among genotypes.

The minor allele frequencies of SNP37_*ICAM1* were 0.31 and 0.38 in the Hyogo and Miyazaki populations, respectively. This SNP was most significant (*p* = 3.33 × 10^−4^) in the Miyazaki population and showed a weakly significant association (*p* = 0.043) in the Hyogo population. Comparing the least-squares means among genotypes, a significant difference was detected between AA and AG in the Miyazaki population, suggesting that animals with the G allele have higher BMS scores than those with the A allele. In Hyogo population, no significant differences were observed among genotypes.

## 4. Discussion

We investigated the effects of 96 candidate polymorphisms on the BMS in 441 Japanese Black cattle in Hyogo. Of the 78 SNPs that were successfully genotyped, 48 were significantly associated with the BMS. In our previous study, one SNP, the p.Lys81Met polymorphism of *SLC27A6*, was verified as a candidate polymorphism for beef marbling. However, in the current study, 78 SNPs were verified simultaneously, and 4 polymorphisms, namely, an SNP in the 3′-untranslated region (UTR) of *LYL* (*p* = 6.83 × 10^−5^), an SNP in the 3′-UTR of *NACC1* (*p* = 6.83 × 10^−5^), a missense polymorphism of *ALDH7A1* (*p* = 3.75 × 10^−5^), and a splice region of *ALDH7A1* (i.e., SNP93_*ALDH7A1*; *p* = 3.46 × 10^−5^), were more strongly associated with the BMS than p.Lys81Met of *SLC27A6*. These results demonstrate that the IFC is a powerful tool in verifying a large number of candidate SNPs.

When the genotypes of SNP93_*ALDH7A1* were considered as fixed effects, the significance of almost all SNPs in the assessed region decreased, indicating that there was a single QTL in the region and that a QTN would be in LD with SNP93_*ALDH7A1*. Further analysis of the LD block region containing SNP93_*ALDH7A1* will likely lead to identification of QTGs and QTNs. However, LD block analysis indicated that it was not possible to determine the range of the LD block in the Hyogo population because a strong LD relationship was found throughout the candidate region (10–30 Mbp). For example, a moderate LD relationship (*r*^2^ = 0.37) was observed between SNP37_*ICAM1* (16.05 Mbp) and SNP93_*ALDH7A1* (28.59 Mbp), even though they are >10-Mbp apart. Therefore, it was necessary to condense the candidate area by conducting similar verification analysis using another Japanese Black population and comparing the LD block results. Accordingly, we also investigated the effects of these SNPs in the candidate area on Miyazaki cattle.

In the Miyazaki population, 76 SNPs were successfully genotyped, and SNP37_*ICAM1*, a missense polymorphism (p.Ala332Thr) of *ICAM1*, was the most significant SNP. In contrast, the most significant SNP in Hyogo cattle, SNP93_*ALDH7A1*, was not significant in Miyazaki cattle. Moreover, SNP37_*ICAM1* was only weakly significant in the Hyogo population compared with the strong association of other SNPs, including SNP93_*ALDH7A1*, suggesting that SNP37_*ICAM1* was unlikely to be in strong LD with QTNs in Hyogo cattle. Overall, these results suggest that each population likely has different QTLs for beef marbling in the assessed regions. In previous studies, the assessed region has been reported as a QTL for BMS-related traits; therefore, it is considered to be derived from the same QTN. However, our results revealed that there were at least two different QTNs depending on the cattle population. The effects of QTLs on the BMS are expected to be clarified by identifying the responsible QTN in each population.

Identifying the QTN in the Hyogo population will be challenging because, in addition to the wide LD range, the most significant SNP, SNP93_*ALDH7A1*, is located near the end of the candidate region (28.6 Mbp); therefore, the peak significance of the SNPs is unclear. In further research, it will be important to condense the candidate region by further expanding the target region, especially downstream of the 30-Mbp region, and increasing the number of markers used for verification. In the Miyazaki population, the LD relationship among SNPs in the assessed region was weaker than that in the Hyogo cattle, and an LD block containing SNP37_*ICAM1* had a range of 15.8–16.1 Mbp, suggesting that QTNs would be located within the region. Among 19 genes that were located in the region (Supplementary Table S2), the function of the *ICAM1*, which contains the most significant SNP and is the most promising candidate QTG, and its effect on the BMS are discussed below.

The *ICAM1* protein is a member of the immunoglobulin superfamily; specifically, *ICAM1* encodes a cell surface glycoprotein that is typically expressed in endothelial cells and immune system cells [11]. *ICAM1* mediates cellular interactions by binding to its counter–receptors and promotes leukocyte migration across the vascular endothelium in processes such as extravasation and the inflammatory response [12,13,14,15]. In addition to functions in the immune system, *ICAM1* is expressed in a variety of cells and plays an important role in adipocyte differentiation.

The CCAAT enhancer binding protein (*C/EBP*) family contains critical transcription factors that function with adipogenic genes [16]. For example, *C/EBPβ* is expressed in preadipocytes to induce adipocyte differentiation [17], and its transcriptional activity is regulated by members of the mitogen-activated protein kinase (MAPK) family, e.g., extracellular signal-regulated kinase (ERK) [18] and p38 MAP kinase (p38) [19]. In short, MAPKs phosphorylate *C/EBPβ* to enhance its transcriptional activity in relation to gene expression was required for cell differentiation into adipocytes. This functional relationship implies that MAPKs, especially ERK and p38, are important factors for adipogenesis.

Xu et al. found that overexpression of *ICAM1* in vitro activated ERK and p38 in human mesenchymal stem cells (MSC), suggesting that *ICAM1* is involved in cell differentiation [20]. Merrick et al. found that *ICAM1* is highly expressed in mouse and human preadipocytes in vivo [21]. The latter authors demonstrated that *ICAM1* expression was increased only at the preadipocyte stage during the differentiation of MSCs into adipocytes, with low expression observed in interstitial progenitor cells and mature adipocytes. On the basis of these previous findings, *ICAM1* likely enhances adipogenesis via activation of ERK and p38 in preadipocytes, resulting in an increase in mature adipocytes.

In summary, *ICAM1* likely plays an important role in adipose differentiation and may affect the expansion of adipose tissue. SNP37_*ICAM1*, which had the most significant association with the BMS of the candidate SNPs, causes an amino acid substitution from alanine to threonine; therefore, SNP37_*ICAM1* could act as a responsible polymorphism in beef marbling by altering the formation and function of the *ICAM1* protein. Additional research on the effects of this missense polymorphism on the *ICAM1* protein and determination of how it affects adipocyte differentiation will help elucidate the mechanism underlying beef marbling formation.

## 5. Conclusions

In two Japanese Black cattle populations, we examined a number of candidate SNPs for a previously reported QTL to identify QTGs and QTNs. Our results suggested that each population likely has a different QTL, even though they are the same breed. Furthermore, in one population, we identified a promising QTG and QTN for beef marbling. Further detailed analysis is needed to identify the QTG and QTN for the QTL in the other population; nevertheless, our findings improve our understanding of the QTLs for beef marbling in beef cattle breeds.

## Figures and Tables

**Table 1:** Effect of SNP93_*ALDH7A1* on BMS in two Japanese Black populations.

Population	N	Genotype Frequency (the Number of Animals)			Allele Frequency		BMS (Least Square Mean) ± SE			*p*-Value
		AA	AC	CC	A	C	AA	AC	CC	
Hyogo	441	0.03	0.42	0.55	0.24	0.76	6.86 ^a^	5.92 ^a^	5.25 ^b^	3.75 × 10^−5^
		(15)	(184)	(242)			±0.45	±0.17	±0.15	
Miyazaki	529	0.01	0.24	0.74	0.13	0.87	6.50	6.30	6.07	0.459
		(6)	(129)	(394)			±0.80	±0.22	±0.16	

^a,b^: means with different superscript are significantly different between genotypes.

**Table 2:** Effect of SNP37_*ICAM1* on BMS in two Japanese Black populations.

Population	N	Genotype Frequency (the Number of Animals)			Allele Frequency		BMS (Least Square Mean) ± SE			*p*-Value
		AA	AG	GG	A	G	AA	AG	GG	
Hyogo	441	0.46	0.47	0.07	0.69	0.31	5.35	5.63	6.17	0.043
		(202)	(208)	(31)			±0.17	±0.16	±0.33	
Miyazaki	529	0.34	0.56	0.10	0.62	0.38	5.64 ^b^	6.37 ^a^	6.05 ^ab^	3.33 × 10^−4^
		(179)	(297)	(53)			±0.20	±0.17	±0.30	

^a,b^: means with different superscript are significantly different between genotypes.

## Data Availability

Not applicable.

## Supplementary Materials

The following supporting information can be downloaded at: https://www.mdpi.com/article/10.3390/genes13071190/s1, Table S1: information of 96 selected SNPs and genotyping results in two cattle populations; Table S2: list of the genes that were located within the candidate region (15819376–16114288 bp) on BTA7.
